# Applications of ChatGPT in the diagnosis, management, education, and research of retinal diseases: a scoping review

**DOI:** 10.1186/s40942-024-00595-9

**Published:** 2024-10-17

**Authors:** Victor C. F. Bellanda, Mateus Lins dos Santos, Daniel Araujo Ferraz, Rodrigo Jorge, Gustavo Barreto Melo

**Affiliations:** 1 Ribeirão Preto Medical School, University of São Paulo, 3900 Bandeirantes Ave, Ribeirão Preto, SP 14049-900 Brazil; 2 Sergipe Eye Hospital, Aracaju, SE Brazil; 3 D’Or Institute for Research & Education, Rio de Janeiro, RJ Brazil; 4 Paulista School of Medicine, Federal University of São Paulo, São Paulo, SP Brazil

**Keywords:** Retinal diseases, Artificial intelligence, Education, medical, Education, patient, Ophthalmology, Decision support systems, clinical, Automation, Health information systems

## Abstract

**Purpose:**

This scoping review aims to explore the current applications of ChatGPT in the retina field, highlighting its potential, challenges, and limitations.

**Methods:**

A comprehensive literature search was conducted across multiple databases, including PubMed, Scopus, MEDLINE, and Embase, to identify relevant articles published from 2022 onwards. The inclusion criteria focused on studies evaluating the use of ChatGPT in retinal healthcare. Data were extracted and synthesized to map the scope of ChatGPT’s applications in retinal care, categorizing articles into various practical application areas such as academic research, charting, coding, diagnosis, disease management, and patient counseling.

**Results:**

A total of 68 articles were included in the review, distributed across several categories: 8 related to academics and research, 5 to charting, 1 to coding and billing, 44 to diagnosis, 49 to disease management, 2 to literature consulting, 23 to medical education, and 33 to patient counseling. Many articles were classified into multiple categories due to overlapping topics. The findings indicate that while ChatGPT shows significant promise in areas such as medical education and diagnostic support, concerns regarding accuracy, reliability, and the potential for misinformation remain prevalent.

**Conclusion:**

ChatGPT offers substantial potential in advancing retinal healthcare by supporting clinical decision-making, enhancing patient education, and automating administrative tasks. However, its current limitations, particularly in clinical accuracy and the risk of generating misinformation, necessitate cautious integration into practice, with continuous oversight from healthcare professionals. Future developments should focus on improving accuracy, incorporating up-to-date medical guidelines, and minimizing the risks associated with AI-driven healthcare tools.

**Supplementary Information:**

The online version contains supplementary material available at 10.1186/s40942-024-00595-9.

## Introduction

Large language models (LLMs) such as ChatGPT (OpenAI, San Francisco, CA, USA), Bing Chat (Microsoft Corporation, Redmond, WA, USA), and Gemini (Google LLC, Mountain View, CA, USA) have gained substantial popularity and represent significant advancements in natural language processing. These models, trained on extensive datasets, can interpret texts, commands, and questions, generating responses that closely mimic human conversation. Among them, ChatGPT has emerged as the most prominent, receiving widespread usage and recognition, even within scientific literature [1]. 

GPT stands for “Generative Pre-trained Transformer,” a type of artificial intelligence model designed to understand and generate human-like text. It is pre-trained on vast amounts of data and fine-tuned for specific tasks, enabling it to respond coherently to a wide range of language inputs. The potential applications of ChatGPT in various fields, including journalism, marketing, education, and professional writing, are considerable. However, its role in medicine, particularly in specialized areas like retinal care, remains uncertain. In medicine, potential uses include medical education, clinical decision support, patient counseling, academic writing, charting, and billing, among others, as the technology continues to evolve [2]. Epic (Epic Systems, Verona, WI, USA), a widely used electronic charting system, has already integrated AI tools to assist with various tasks such as generating visit summaries, coding, drafting messages to patients, and charting. These AI-driven features aim to streamline administrative tasks, reduce the burden on healthcare providers, and improve the overall efficiency of patient care [3]. 

One of the most explored applications of ChatGPT in ophthalmology is medical education. Studies have shown that ChatGPT often outperforms other LLMs in medical knowledge assessments, including board-style questions, official board examinations, and challenging clinical cases, sometimes even surpassing the performance of the average physician [4–13]. Although caution is needed due to the potential for inaccuracies, ChatGPT shows significant promise as a supportive tool throughout various stages of medical training.

In patient management, the performance of ChatGPT has yielded mixed results. It is possible to obtain diagnostic and management recommendations by inputting patient data and clinical presentations, but concerns about hallucinations—where the AI generates plausible-sounding but factually incorrect or nonsensical information—and factual inaccuracies limit its reliability. For example, ChatGPT may fabricate references that do not exist, presenting them as legitimate sources of information [14]. Studies have evaluated ChatGPT’s ability to provide management advice, with results varying based on case complexity, presentation style, and the benchmarks used for comparison [14–25]. The use of ChatGPT for triage and direct patient counseling in ophthalmology and retinal care has also been investigated. Patients could interact with the LLM to describe their symptoms and receive guidance on the appropriate level of care. However, challenges such as potential patient miscommunication and the LLM’s ability to accurately interpret symptoms remain [26–28]. 

Another area where ChatGPT could be beneficial is in automating charting and billing processes. In versions that accept audio inputs, ChatGPT can process clinical consultation data to generate organized reports and notes, potentially reducing paperwork and freeing resources for other aspects of medical care [29–33]. Nevertheless, concerns about inaccuracies, privacy, and security are significant, particularly regarding the sensitivity of patient information and evolving regulations on data privacy for AI tools [33]. 

There has also been significant discussion about ChatGPT’s role in academic writing. For non-native English speakers, ChatGPT offers considerable benefits as a tool for drafting and reviewing academic content [34–36]. However, its tendency to produce hallucinations and factual errors raises concerns about academic integrity. Issues related to authorship and responsibility have been highlighted, with a consensus that ChatGPT should not be credited as an author in most scientific journals [37–39]. 

To better understand ChatGPT’s applications in retinal care, we conducted a scoping review of the literature. Our review highlights the primary uses of this LLM in the retina field, along with the associated risks and challenges it presents.

## Methods

This scoping review aimed to comprehensively explore the current literature on the applications of ChatGPT in the diagnosis, management, and research of retinal diseases. Prior to initiating the review, we conducted a preparatory literature search to ensure no similar reviews had previously been conducted. To maintain high standards of scientific rigor, we published a prospectus of our review on the Open Science Foundation (OSF) public repository. The original prospectus can be found in the supplementary materials. The review protocol adhered to the PRISMA (Preferred Reporting Items for Systematic reviews and Meta-Analyses) guidelines for scoping reviews [40]. 

We executed a comprehensive search across multiple databases, including PubMed, Scopus, MEDLINE, and Embase, to identify relevant articles. Our search strategy comprised specific query strings designed to capture articles discussing the use of ChatGPT in retinal healthcare. For the search to be comprehensive yet focused, we stipulated that each article must contain at least one keyword from both of our designated groups. Group 1 keywords are centered around the technology in question: ChatGPT, GPT, LLM, or “large language”. Group 2 keywords pivot around the field of retinal diseases: ophthalmolog*, retin*, vitre*, uvea*, uvei*, chor*, and macul*. Initially, “eye” was a part of Group 2, and “generative” was included in Group 1. However, upon review, “eye” was casting too wide a net, capturing an excess of articles not specific to ophthalmology. Similarly, “generative” was drawing in a significant number of irrelevant results related to generative adversarial networks, rather than our intended AI models. Therefore, we refined our terms to the most pertinent and specific to our research. The detailed search strings used in each database are presented in Table 1. In addition to database searches, we performed hand-searching of reference lists and grey literature using web-based search engines such as Google Scholar and repositories like ResearchGate. Preprints available in Scopus were also included. Given that the first version of ChatGPT was launched in November 2022, the search period was restricted to publications from that year onward. The searches were conducted on April 21, 2024. To ensure the inclusion of recent literature, a simplified search was conducted at the end of the review process, two months later, focusing on the terms “ChatGPT” and “retina,” and was limited to PubMed.

**Table 1:** – search queries per database

Database	Search query	Number of Results
PubMed	((“ChatGPT“[All Fields] OR “GPT“[All Fields] OR “LLM“[All Fields] OR “large language“[All Fields]) AND (“ophthalmologie“[All Fields] OR “ophthalmology“[MeSH Terms] OR “ophthalmolog*“[All Fields] OR “ophthalmology s“[All Fields] OR “retin*“[All Fields] OR “vitre*“[All Fields] OR “uvei*“[All Fields] OR “uvea*“[All Fields] OR “chor*“[All Fields] OR “macul*“[All Fields]) AND 2022/01/01:2024/12/31[Date - Publication]) AND (2022/1/1:2024/12/31[pdat])	197
Scopus	TITLE-ABS-KEY ( {ChatGPT} ) OR TITLE-ABS-KEY ( {GPT} ) OR TITLE-ABS-KEY ( {LLM} ) OR TITLE-ABS-KEY ( {large language} ) AND TITLE-ABS-KEY ( ophthalmology ) OR TITLE-ABS-KEY ( retin* ) OR TITLE-ABS-KEY ( vitre* ) OR TITLE-ABS-KEY ( uvea* ) OR TITLE-ABS-KEY ( uvei* ) OR TITLE-ABS-KEY ( chor* ) OR TITLE-ABS-KEY ( macul* ) AND PUBYEAR > 2021 AND PUBYEAR < 2025 AND ( LIMIT-TO ( LANGUAGE, “English”))	160
MEDLINE	(chatgpt OR gpt OR “large language”) AND (ophthalmolog* OR retin* OR uvea* OR uvei* OR chor* OR vitre* OR macul*) AND ( la: (“en” OR “de” OR “es”)) AND (year_cluster: [2022 TO 2024])	136
Embase (Elsevier)	((ChatGPT OR GPT OR LLM OR ’large language’) AND (ophthalmology/exp OR ophthalmolog* OR retin* OR vitre* OR uvei* OR uvea* OR chor* OR macul*) AND 2022/de) AND (2022/de)	25

*Each article may have been assigned to one or more categories

The inclusion criteria for this review encompassed studies discussing the application of ChatGPT across all aspects of retinal healthcare and knowledge production, involving researchers, healthcare professionals, patients, and their families within the care setting for retinal diseases. We concentrated on studies evaluating the feasibility, task facilitation, accuracy, and limitations of using ChatGPT. Articles were excluded if they did not pertain to retinal diseases, assessed other forms of AI without direct comparison to ChatGPT, were purely theoretical without empirical evaluation or application, or were not available in English.

Two independent, blinded reviewers (VCFB and MLS) screened the titles and abstracts of identified studies for eligibility using the Rayyan platform (Rayyan Inc, USA). Full texts of potentially eligible studies were retrieved and independently assessed. Any disagreements were resolved by consensus, ensuring a rigorous inclusion of relevant articles.

Data extraction was conducted by the two independent reviewers, who documented study characteristics, application areas, and outcomes from each eligible study. Studies were categorized based on their practical application areas, with the possibility of a single article falling into multiple categories. These categories included: academics and research, charting, coding and billing, diagnosis, disease management, literature consulting, medical education, and patient counseling. Outcomes of interest included the feasibility of using ChatGPT, its effectiveness in facilitating healthcare-related tasks, the accuracy of the information provided, and the limitations or challenges encountered in its application.

The extracted data were synthesized narratively to map the scope of ChatGPT’s applications in retinal healthcare. We identified patterns, themes, potentials, and limitations across different application areas.

As this study was a scoping review, it did not involve direct interaction with human subjects, and therefore, ethical approval was not required. Nevertheless, the review was conducted following rigorous scientific and ethical standards to ensure the reliability and validity of the findings.

## Results

The initial search conducted on April 21, 2024, yielded a total of 518 articles. The distribution of these articles across the different databases was as follows: PubMed (197), Scopus (160), MEDLINE (136), and Embase [25]. After removing 256 duplicates, 262 unique articles remained for screening.

During the independent screening process, the reviewers initially disagreed on the inclusion of 10 articles. These conflicts were resolved through open discussion, resulting in 98 articles being included for full-text reading. Following detailed examination, 32 articles were excluded for not meeting the inclusion criteria, leaving a total of 66 articles for inclusion in the final analysis.

**Fig. 1:**
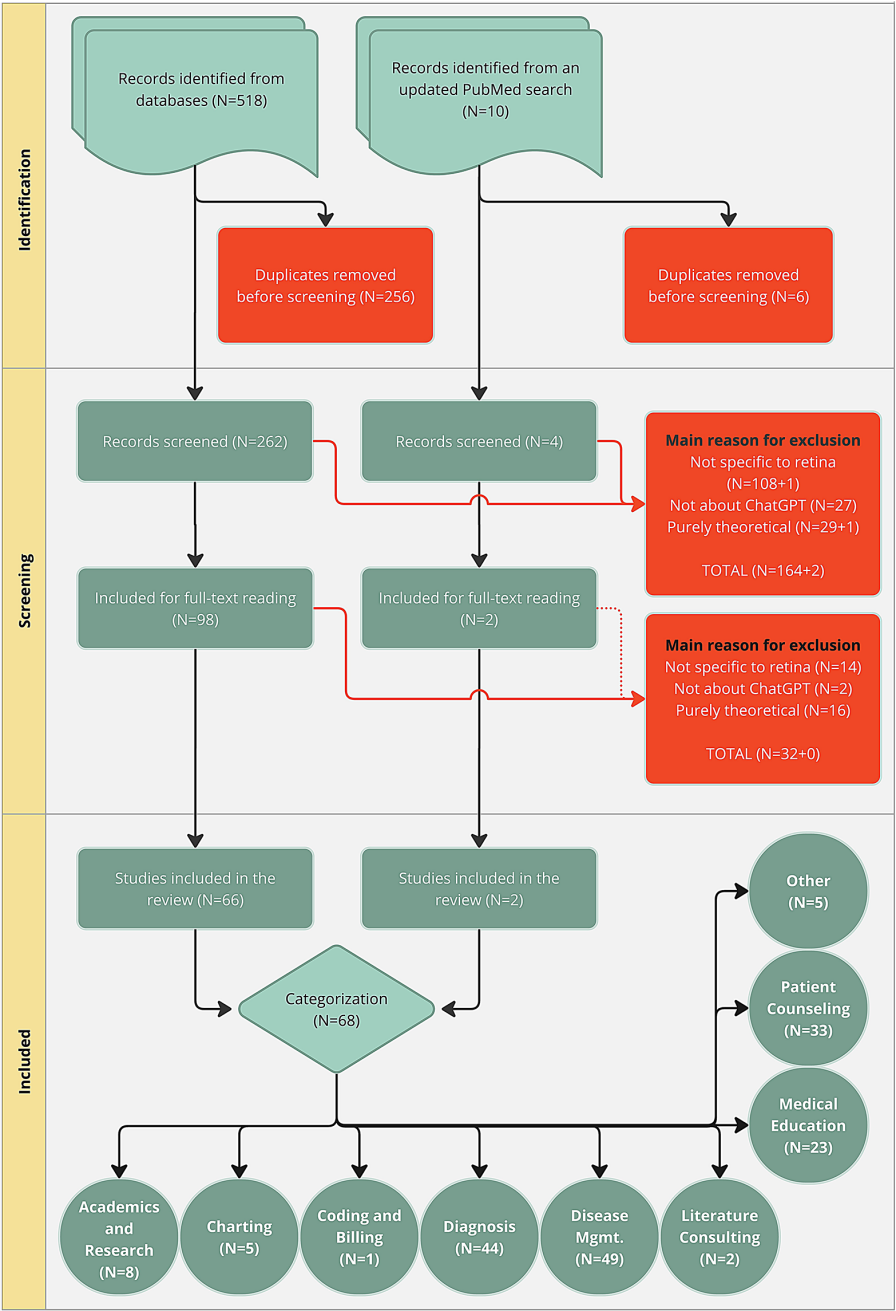
PRISMA flowchart

Out of the 164 articles excluded during the screening phase, 108 were not specific to the retina, 27 did not pertain to ChatGPT, and 29 were classified as purely theoretical, including review articles, comments (without empirical data), and replies. From the 98 articles assessed for full-text eligibility, an additional 32 were excluded: 14 were not specific to retina, 2 were not about ChatGPT, and an additional 16 were purely theoretical. It is important to note that articles not specific to retina but related to ophthalmology in general were included if they compared subspecialties and/or specifically mentioned retina. A PRISMA flowchart is presented in Fig. 1.

To ensure the inclusion of the most recent and relevant studies, an update query was conducted on July 7, 2024, using PubMed with the search terms “ChatGPT” and “retina,” filtered for publications from April 2024 onwards. This search resulted in 10 additional articles, of which 6 were identified as duplicates. After reviewing the full text of the remaining 4 articles, 2 were deemed relevant and included in the analysis. The other 2 were excluded due to being purely theoretical and not related to retinal care, respectively.

In total, 68 articles were included in this scoping review. Although purely theoretical articles, such as commentaries and reviews, were excluded from the primary analysis, they were taken into consideration when deemed relevant (i.e., if they introduced new and pertinent information). This approeach ensured a comprehensive overview of the current state of knowledge and opinions regarding the use of ChatGPT in retinal healthcare.

**Fig. 2:**
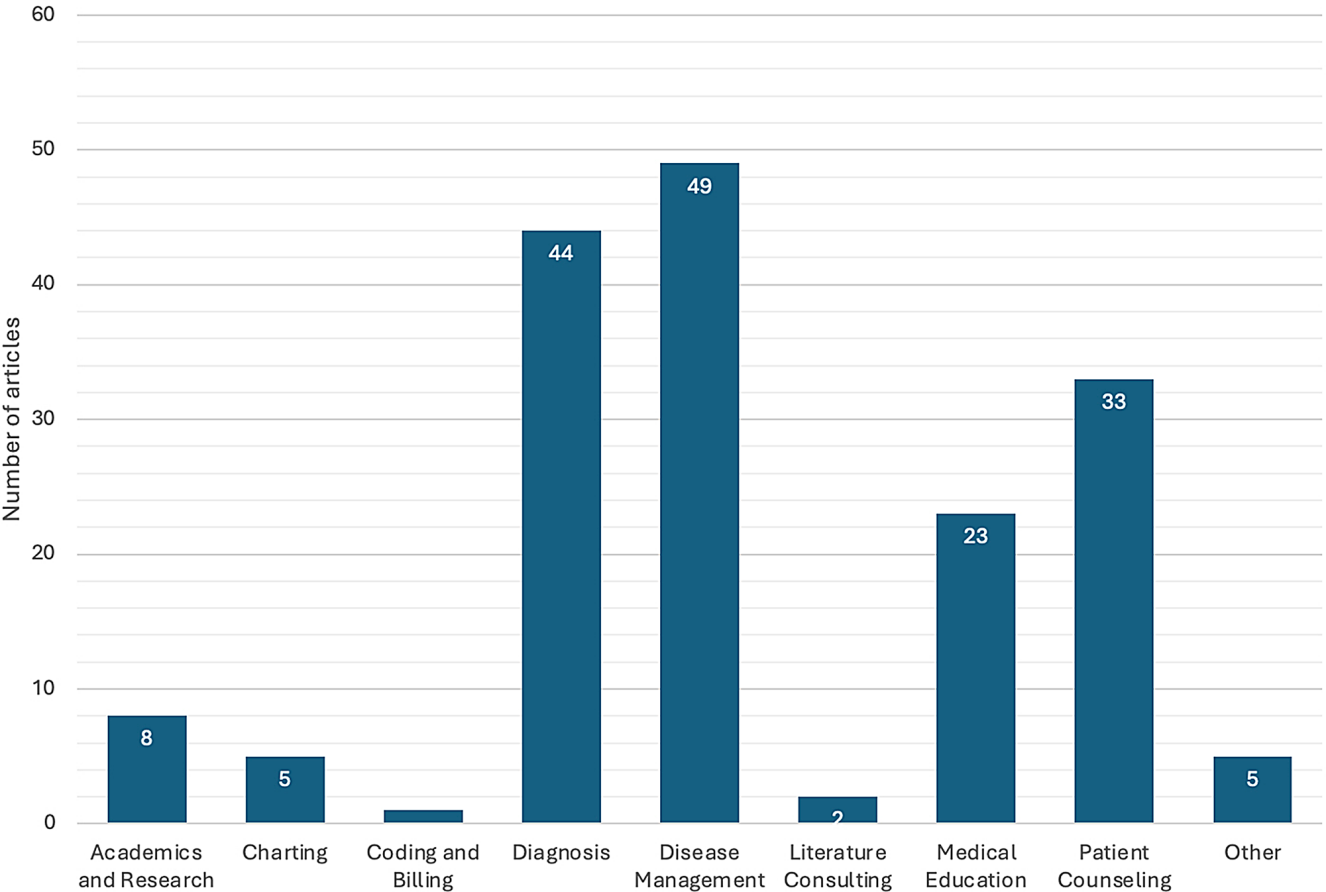
Number of articles per category*. *Each article may have been assigned to one or more categories

The articles were categorized as follows (Fig. 2): 8 focused on academics and research, 5 on charting, 1 on coding and billing, 44 on diagnosis, and 49 on disease management. Additionally, 2 articles were about literature consulting, and 23 addressed medical education, specifically analyzing ChatGPT’s performance on exams. Furthermore, 33 articles involved patient counseling, either in preparing patient materials or evaluating ChatGPT’s reliability as an information source. Lastly, 3 articles dealt with symptom triaging, a category not initially defined in the protocol. During screening and extraction, this category was temporarily labeled as “Other” but was later redefined as “Triage and Pre-Hospital Management” for better organization. This category includes the processes involved in the initial assessment and prioritization of patients’ symptoms, as well as decisions regarding the need for immediate medical intervention before hospital admission.

## Discussion

### Academics and research

The integration of ChatGPT and other AI tools into academic and research practices within retinal care showcases both promising potential and significant limitations. Various studies have explored the utility of these technologies in different capacities, providing insights into their performance and highlighting areas for improvement.

From the studies focused on the academic uses of ChatGPT, the authors highlight the one published by Valentín-Bravo et al., who examined ChatGPT for its ability to generate scientific content on topics such as the complications associated with silicone oil in vitreoretinal surgery. While ChatGPT was capable of producing coherent summaries, it often lacked the necessary scientific rigor and accuracy, exhibiting issues such as inappropriate scientific discourse and incorrect bibliographic references. This underscores the necessity of human oversight to ensure reliability and address ethical concerns [41]. Comparatively, the performance of ChatGPT-3.5 and Bing Chat on a multiple-choice ophthalmology exam revealed that Bing Chat, enhanced by its integration with online data retrieval, outperformed ChatGPT-3.5. However, the reliance on non-peer-reviewed sources by Bing Chat necessitated a careful appraisal of its responses for educational purposes, highlighting the importance of verifying the quality and reliability of AI-generated content [42].

Further illustrating AI’s potential, the development of a bilingual Chinese-English Indocyanine Green Angiography (ICGA) report generation and QA system using AI demonstrated high accuracy and substantial agreement among ophthalmologists. Nevertheless, the system struggled with rare cases and occasionally provided vague or inaccurate answers. This suggests that while AI can streamline research processes, particularly in managing large datasets and facilitating communication across languages, there remains a need for ongoing optimization [43]. However, there may be a gap in ChatGPT’s performance across different languages. In a study on retinal vascular disease classification, ChatGPT’s diagnostic performance was superior with English prompts compared to Chinese prompts, pointing to the limitations of current large language models in non-English settings and the need for further development [44].

In more complex clinical scenarios, GPT-4’s performance in answering questions about intricate ophthalmology cases was evaluated. An article by Milad et al. demonstrated that improved prompting strategies enhanced GPT-4’s performance, yet it still lagged behind ophthalmology trainees. This article, however, introduced valuable concepts on how proper prompt engineering can yield better results. It compares Traditional Zero-Shot Prompting, which involves directly asking GPT-4 to solve a task without providing any prior examples or structured guidance, with Zero-Shot Plan-and-Solve+ (PS+). The PS + strategy involves asking GPT-4 to create a plan by breaking down the main task into smaller subtasks, followed by executing these subtasks with detailed instructions. This method improved the logical reasoning and accuracy of the model by providing a structured approach to problem-solving [11].

To ensure more reliable responses, a group of researchers investigated the integration of GPT-4 with verified textbook knowledge. For this purpose, they created Aeyeconsult, an AI chatbot that leverages verified textbook knowledge alongside GPT-4. Aeyeconsult demonstrated superior accuracy and consistency compared to ChatGPT-4 alone. The use of verified sources and citation provision significantly enhanced the reliability of Aeyeconsult, underscoring the importance of source verification in AI applications for academic purposes and suggesting a pathway to improve AI reliability in medical education and research [45].

The evaluation of self-awareness capabilities in ChatGPT and its competitor Google Bard, now Google Gemini (Google Inc, Mountain View, CA, USA), found that while these chatbots showed some ability to self-correct, their self-checking capabilities were limited. This highlights the need for continuous improvement in AI’s ability to autonomously verify and correct its outputs, a vital feature for ensuring the reliability of AI-generated academic content [22].

Mihalache et al. evaluated the performance of ChatGPT-3.5 and ChatGPT-4 in providing information on age-related macular degeneration (AMD) and diabetic retinopathy (DR), comparing the chatbot responses with the American Academy of Ophthalmology’s Preferred Practice Pattern guidelines. Both models performed similarly, generally providing accurate and readable responses, though caution is advised as occasional inaccuracies and omissions were observed, emphasizing the need for professional oversight [46].

Lastly, in another investigation, Taloni et al. explored the potential misuse of ChatGPT-4’s Advanced Data Analysis (ADA) capabilities to create a fake medical dataset. Researchers provided the model with detailed prompts to fabricate data for 300 eyes from 250 keratoconus patients who underwent deep anterior lamellar keratoplasty (DALK) or penetrating keratoplasty (PK). The results showed that ADA successfully created a seemingly authentic dataset with statistically significant differences in preoperative and postoperative outcomes favoring DALK over PK. While not directly related to retinal care, this study raises significant concerns about the potential for ADA to generate convincing but false scientific evidence. This underscores the necessity for robust strategies to detect AI-generated data fabrication and safeguard the integrity of scientific research, whether in retinal ophthalmology or other fields of science [47].

In summary, while ChatGPT and similar AI tools hold significant potential for advancing academic and research activities in retinal care, their current limitations necessitate a cautious and critical approach. Ensuring human oversight, source verification, and addressing ethical concerns are imperative steps in harnessing the full potential of AI in this field.

### Charting

The application of ChatGPT and other LLMs in the field of retina care extends significantly into the domain of charting, which includes tasks such as describing patient encounters, producing surgical notes, and creating discharge summaries. Various studies have highlighted both the potential benefits and limitations of these AI tools in automating and enhancing documentation processes [29–32, 43].

In one study, researchers developed a system called ICGA-GPT to assist with the interpretation of ICGA images by automating the generation of bilingual reports and enabling interactive question-answering. The ICGA-GPT model demonstrated satisfactory performance in generating detailed reports, with substantial agreement among ophthalmologists on their completeness and accuracy. This capability can significantly reduce the time and effort required by ophthalmologists write ancillary examination reports and explain them to patients, thereby improving workflow efficiency and patient care [43].

The use of GPT-4 in documenting surgical notes for ophthalmic procedures has also shown promise. Studies have explored its application in generating detailed and contextually accurate operative notes for cataract surgeries, including complex cases with complications. This capability suggests potential applications in documenting retinal procedures as well. Furthermore, the ability of GPT-4 to generate templates without inputting confidential medical information ensures patient privacy while maintaining efficiency [29, 30].

Further extending the capabilities of ChatGPT, another study examined its performance in generating discharge summaries and operative notes across various ophthalmic subspecialties. ChatGPT produced valid and detailed documents rapidly, with the ability to incorporate specific medications, follow-up instructions, and other essential details based on the quality of input prompts. However, the presence of generic text and occasional factual inaccuracies necessitated human verification. The ability of ChatGPT to admit mistakes and correct itself upon prompting highlights its potential for continuous learning and improvement [31].

In summary, while ChatGPT and similar LLMs demonstrate significant potential in automating and enhancing charting tasks in retina care, their current limitations necessitate cautious integration into clinical practice. Ensuring human oversight, continuous updates, and validation by healthcare professionals are crucial to harness the full benefits of these AI tools. Future developments should focus on improving accuracy, incorporating up-to-date medical guidelines, training to minimize generic text and progressively replace it with customized, patient-specific information, and enhancing the models’ ability to autonomously verify and correct their outputs. With these improvements, AI can play a transformative role in reducing the administrative burden on healthcare providers and improving the overall efficiency and quality of medical documentation.

### Coding and billing

The application of ChatGPT and other LLMs in coding and billing within retina care presents a promising opportunity to reduce administrative burdens. Ong et al. evaluated ChatGPT’s ability to generate ICD codes for retina clinic encounters, a crucial yet time-consuming task for physicians. Retina specialists created mock clinic scenarios, which were inputted into ChatGPT to generate the appropriate ICD codes. ChatGPT correctly produced ICD codes for 70% of the encounters, demonstrating a notable true positive rate. However, the study also identified significant weaknesses, such as the generation of fabricated or incorrect codes and occasional “hallucinations” of plausible-sounding but incorrect responses [32]. These findings highlight the need for improvements, including feedback fine-tuning and updated ICD code guidelines, to enhance accuracy and reliability in clinical practice.

### Diagnosis

The integration of LLMs like ChatGPT into ophthalmology has demonstrated both significant promise and notable challenges, as highlighted by various studies exploring their application in the diagnostic process of ophthalmic conditions. These studies underscore advancements in AI models, particularly in improving diagnostic accuracy, while also identifying areas that require further refinement.

In terms of performance on diagnostic examinations, several studies have shown that newer iterations of LLMs, such as GPT-4, exhibit enhanced capabilities compared to earlier versions like GPT-3.5. For instance, Lin JC and colleagues reported that GPT-4 outperformed both GPT-3.5 and human participants on a practice ophthalmology written examination, scoring 76.9%, compared to 63.1% for GPT-3.5 and 72.6% for humans [48]. This trend of improved performance was echoed by Teebagy S. et al., who found that GPT-4 achieved a higher score on the Ophthalmology Knowledge Assessment Program (OKAP) examination, correctly answering 81% of the questions versus 57% for GPT-3.5 [49]. These findings suggest that the architectural advancements and enhanced training data in GPT-4 significantly contribute to its superior diagnostic performance.

Shemer et al. evaluated the diagnostic accuracy of ChatGPT in ophthalmology by comparing it to residents and attendings. ChatGPT showed lower accuracy rates (54–68%) compared to residents (75–94%) and attendings (71–86%), but it was significantly faster in providing diagnoses, completing cases in a fraction of the time required by humans [50].

Further research by Alexander AC et al. highlighted GPT-4’s superior handling of complex medical knowledge, achieving a perfect score on multiple-choice questions from India’s Foreign Medical Graduate Examination (FMGE), showcasing its potential in diagnostic applications [12]. Similarly, Antaki F et al. observed that GPT-4’s performance on ophthalmology board-style questions was on par with human respondents [5].

Beyond performance on exams, studies have also assessed ChatGPT’s diagnostic accuracy and its utility in clinical decision-making. For example, Hu X et al. investigated GPT-4’s ability to diagnose rare ophthalmic diseases and found that it was most accurate in scenarios where detailed clinical information was available, suggesting its potential as a consultation tool [51]. On the other hand, Haddad F et al. noted that while ChatGPT’s diagnostic accuracy did not surpass that of human experts in answeting ophthalmology-related questions, it still holds strong potential as an educational resource [10]. 

However, limitations remain. Raghu et al. evaluated ChatGPT’s potential as a tool for assessing diabetic retinopathy (DR) risk by analyzing clinical and biochemical data from 111 diabetic patients. While ChatGPT demonstrated good reliability in its responses, its sensitivity and specificity in predicting DR were moderate, indicating further optimization is needed before it can be reliably used in clinical settings [52]. Cai LZ et al. pointed out that while GPT-4 performed comparably to the average human respondent on ophthalmology board-style questions, it still requires improvements in clinical decision-making and diagnostic recommendations [7]. Similarly, Jiao C et al. demonstrated that GPT-4 outperformed GPT-3.5 in addressing multiple-choice ophthalmic case challenges, with improved accuracy even in image-related questions, but acknowledged the the risk of generating fictional information and weakness in more complex inference-based cases [8].

Milad D et al. assessed GPT-4’s ability to diagnose and recommend treatment for complex ophthalmology cases, noting that enhanced prompting strategies improved its performance, though it still did not surpass human expertise [11]. So called “prompt engineering” enhances GPT’s diagnostic capabilities by optimizing the way information is requested and processed. By carefully crafting prompts to include specific clinical details or guide the AI’s reasoning process, clinicians can improve the accuracy and relevance of GPT’s responses, making it a more effective tool in diagnostic applications [11]. In a comparative study of AI chatbots, Sensoy E et al. found that Bard, Bing, and ChatGPT had no statistically different accuracy in diagnosing ophthalmic pathologies and intraocular tumors [53]. 

The application of AI in ophthalmic imaging has also been explored. Chen X et al. developed a model called “ICGA-GPT”, which showed high specificity and accuracy in generating reports from ICGA images. The model not only generated reports based on the images but also suggested diagnoses and provided interactive question-answering capabilities, offering explanations and clarifications regarding the generated findings. The authors highlight the need for more diverse datasets and better handling of rare conditions, despite the promising initial results [43]. Furthermore, Mihalache A et al. evaluated GPT-4’s ability to interpret ophthalmic images and noted a 70% accuracy rate overall, with the highest accuracy in the retina category. The study emphasizes the challenges AI faces with complex visual data, suggesting that multimodal AI tools could significantly enhance diagnostic capabilities if integrated carefully [54].

Comparative studies between different AI models further illustrate the advancements and limitations of current technologies. Masalkhi M et al. compared Meta’s Llama 2 (Meta Inc, Menlo Park, CA, USA) and ChatGPT, finding that while both models provided appropriate medical advice, they had unique strengths and weaknesses in image analysis and medical insights [55]. Another study by the same group compared Google’s Gemini AI with ChatGPT, highlighting Gemini AI’s notable improvements in image analysis but also its limitations compared to GPT-4 [56].

In specialized applications, Singer MB et al. discussed the development of Aeyeconsult, an AI-powered ophthalmology chatbot that outperformed ChatGPT-4 in accuracy by leveraging verified textbook knowledge. The authors compared responses to ophthalmology questions by Aeyeconsult and ChatGPT-4, finding that Aeyeconsult’s integration of verified sources significantly enhanced its accuracy and reliability [45]. 

Rojas-Carabali W et al. evaluated ChatGPT’s diagnostic accuracy for uveitis, revealing that although the model’s diagnostic suggestions were generally accurate, they did not surpass human accuracy, underscoring the need for human oversight. The authors recommend using specialized uveitis databases to train the model and enhance its performance in domain-specific diagnoses. Interestingly, when prompted for the most likely diagnosis along with the two most probable differential diagnoses, both GPT-3.5 and GPT-4 models showed an 8% increase in diagnostic accuracy [57, 58].

Thirunavukarasu AJ et al. examined the performance of ChatGPT in a general practice setting, demonstrating that GPT-4 showed significant improvements over GPT-3.5, suggesting its potential for integration into clinical practice [59]. In a comparative study, Waisberg E et al. evaluated Google’s AI chatbot Bard against ChatGPT, highlighting Bard’s strength in delivering accurate and specific medical advice, largely due to its internet connectivity. However, they emphasized the necessity for ophthalmology-specific training data to further enhance the model’s effectiveness [60] 

Meanwhile, Tao BK et al. compared the performance of Bing Chat, which also has internet access, with GPT-3.5, which lacks this feature, on a multiple-choice ophthalmology exam. Their findings indicated that Bing Chat’s ability to search the web enhanced accuracy rates, while also providing better readability and more reliable citation features [42]. 

### Disease management

The application LLMs like ChatGPT in disease management within ophthalmology has demonstrated substantial potential, yet it also highlights several areas requiring improvement. The following discussion integrates findings from various studies, emphasizing how ChatGPT can support physicians in decision-making, triage, and evidence-based practice.

Studies by Lin JC et al. and Alexander AC et al. demonstrated GPT-4’s superior performance on practice ophthalmology examinations compared to GPT-3.5 and human users, suggesting its utility in aiding physicians’ understanding of complex medical topics [12, 48]. However, Antaki F et al. highlighted that while GPT-4 showed significant improvement over GPT-3.5 in answering ophthalmology board-style questions, it did not outperform human experts [6]. Cai LZ et al. echoed these findings, noting that GPT-4 performed comparably to the average human respondent but still required further refinement in clinical decision-making and diagnostic recommendations [7]. Generally, ChatGPT performed better on written questions and worse on image-based questions [48].

The application of ChatGPT in clinical decision support has been explored through various studies. Haddad F et al. evaluated ChatGPT’s responses to ophthalmology-related questions and found that while its diagnostic accuracy was commendable, it was lower than that of human experts [10]. Inayat H et al. similarly noted that ChatGPT’s accuracy in determining the degree of urgency of given clinical pictures was lower than that of ophthalmology residents and consultants, emphasizing the need for human involvement in clinical decisions [61]. Carlà MM et al. found that, in comparison with ChatGPT-3.5 and Google Gemini, ChatGPT-4 shows the highest accuracy and agreement with expert surgeons, surpassing both models in offering detailed and coherent surgical plans, particularly in complex cases like combined cataract and retinal surgery [62].

ChatGPT’s role in supporting evidence-based practice has been explored through its ability to provide accurate and reliable medical information. Capellani F et al. evaluated the reliability and accuracy of ChatGPT’s responses on ophthalmic diseases compared to AAO guidelines, finding that while most answers were correct and useful, a significant portion was incomplete or incorrect. This study concluded that ChatGPT should be used with medical supervision to avoid misinformation [14]. As mentioned in the previous section, Sakai D et al. developed Aeyeconsult, an AI-powered ophthalmology chatbot leveraging verified textbook knowledge and GPT-4, which outperformed ChatGPT-4 in accuracy. This study demonstrated the feasibility and enhanced reliability of using AI chatbots that cite verified sources, suggesting significant potential for such tools in clinical practice [63].

While the studies reviewed show that ChatGPT and other LLMs hold significant promise in supporting disease management, several limitations remain. For instance, ChatGPT often provides general rather than specific, contextually relevant information, as noted by Choudhary A et al. [64] Their study compared the treatment recommendations provided by AI platforms, such as ChatGPT 3.5, ChatGPT 4.0, and Bing AI, with those given by retina specialists. The findings revealed moderate to substantial agreement between AI and clinician recommendations, particularly for the management of diabetic macular edema (DME), though there were some discrepancies in cases involving co-existing ocular and systemic co-morbidities. The study concludes that AI has potential as a complementary tool in DME management but emphasizes the need for further refinement to align AI recommendations more closely with clinical guidelines. [64] Additionally, Subramanian B et al. found that while ChatGPT-4 provided highly appropriate and mostly complete answers to queries related to diabetic retinopathy, certain complex medical queries were less comprehensively addressed, indicating the need for ongoing refinement [65].

Milad D et al. assessed GPT-4’s ability to diagnose and recommend treatment for complex ophthalmology cases, noting that enhanced prompting strategies improved its performance. However, GPT-4 was still outperformed by senior ophthalmology residents, suggesting its current limitations in providing expert-level guidance [11]. Maywood et al. assessed the performance of ChatGPT in clinical vitreoretinal scenarios, concluding that while the AI tool can provide accurate responses for straightforward cases, further development is needed to improve its capabilities in more intricate clinical environments [66].

### Literature consulting

The use of ChatGPT and other LLMs in literature consulting shows significant promise for advancing academic research and clinical decision-making in retina care. LLMs can greatly facilitate literature consulting in several ways. Firstly, they can streamline the literature review process by quickly summarizing large volumes of academic papers, identifying key findings, and highlighting relevant studies [67]. This can save researchers considerable time, allowing them to focus on deeper analysis and interpretation. Additionally, LLMs can identify gaps in existing literature and suggest areas for future research by analyzing patterns and trends across numerous publications [68].

GPT models can also facilitate real-time consultation on clinical cases by providing summaries of relevant studies and guidelines [67]. This is particularly beneficial in multidisciplinary team meetings or when seeking second opinions, where quick access to the latest evidence-based information is crucial for informed decision-making. For instance, a clinician could use GPT to retrieve and summarize the latest research on challenging retinal disease cases, treatment options, and outcomes [60]. 

The continuous learning and improvement capabilities of AI models further enhance their utility in literature consulting. As these models are exposed to more data and refined through feedback, their accuracy and relevance in responding to queries improve, leading to highly reliable and personalized literature consulting tools. As previously mentioned, a study by Singer et al. explored the capabilities of a specialized AI chatbot, Aeyeconsult, which integrates GPT-4 with verified ophthalmology textbooks to answer ophthalmology-related questions. Aeyeconsult demonstrated superior performance compared to standard ChatGPT-4, achieving an 83.4% accuracy rate in answering ophthalmology questions, compared to 69.2% for ChatGPT-4 [45].

However, the limitations of GPT and LLMs, such as generating plausible but incorrect information (“hallucinations”) and occasionally providing outdated or irrelevant citations, must be acknowledged [37–39]. Implementing human oversight and validation is crucial to ensure the accuracy and reliability of AI-generated responses, as the responsibility for each case will remain with the assigned ophthalmologist regardless of any content provided by ChatGPT. Recently, a team of researchers from Harvard and MIT developed OpenEvidence (Xyla Inc, Wilmington, DE, USA), an LLM-based chatbot app similar to ChatGPT, but for medical literature consulting. Unlike ChatGPT, OpenEvidence retrieves information exclusively from trusted, peer-reviewed sources. This approach may reduce the risk of hallucinations and inaccurate information often associated with other LLMs, making it more suitable for clinical applications [69].

### Medical education

The “Medical Education” category includes studies that evaluate ChatGPT’s performance in standardized medical exams and educational tools. This section explores ChatGPT’s outcomes in ophthalmology-specific examinations, emphasizing its strengths, areas for improvement, and the ways these models have been integrated into the educational framework for medical practitioners. A total of 23 articles examined the performance of different ChatGPT versions, including GPT-3.5 and GPT-4, on standardized examinations. While ChatGPT performed consistently across studies and frequently attained passing grades in most exams — even surpassing human examinees in some studies — it still has room for improvement, particularly in subspecialty topics such as retinal care. This trend underscores the challenges faced by AI in mastering niche medical knowledge.

Comparative studies highlighted significant improvements in the capabilities of newer models, such as ChatGPT 4.0, over their predecessors, like ChatGPT 3.5 [6, 13]. ChatGPT was tested in multiple board-style and official ophthalmologic knowledge evaluation exams, and the most commonly used test for performance evaluation were the OKAP and American Academy of Ophthalmology’s (AAO) Basic and Clinical Sciences Course’s Self-Assessment Program (BCSC-SAP). Mihalache A et al. tested ChatGPT’s accuracy in January and February of 2023 on practice questions for OKAP. The authors noted that even though results improved over time, with 46% of questions answered correctly in January vs. 58% in February, the performance remained insufficient for board certification preparation [13]. The authors also found that ChatGPT’s performance was much better in broader topics, such as the ones characterized as “General Medicine”, where 79% of answers were correct, against no correct answers in the “Retina and Vitreous” Sect. [13]. These results are in line with the study by Antaki et al., who demonstrated significant improvements in ChatGPT-4.0 over 3.5, especially in clinical reasoning and multi-step practice questions for OKAP, with performance varying by question complexity, attaining better results in simpler questions [6]. Teebagy S et al. noted ChatGPT-4’s superior performance on the OKAP examination compared to ChatGPT-3.5, highlighting its potential in ophthalmologic education and clinical decision support systems [49]. A similar trend was observed by Antaki et al. in OKAP, BCSC-SAP, and OphthoQuestions questions, with ChatGPT 3.5 outperforming the legacy version [5, 6]. Haddad et al. compared ChatGPT’s performance on ophthalmology questions from OKAP and the United States Medical Licensing Examination (USMLE), finding GPT-4.0 significantly outperformed GPT-3.5, and both presented poorer performances when progressing through levels of these exams [10]. 

Cai et al. compared ChatGPT-3.5, ChatGPT-4.0, and Bing Chat on ophthalmology board-style questions from BCSC-SAP, finding ChatGPT-4.0 superior in single-step reasoning but struggling with image interpretation and calculations [7]. Sensoy E et al. also evaluated ChatGPT-3.5, Bing, and Bard in BCSC-SAP questions, focusing on ophthalmic pathology and intraocular tumors. No statistically significant performance differences were found, however, Google Bard presented correct answers to 69.4% of the questions against only 58.6% from ChatGPT-3.5 and 63.9% from Bing [53]. Taloni et al. compared ChatGPT-3.5, ChatGPT-4.0, and average human performance (as provided by AAO) in BCSC-SAP questions as well, finding that ChatGPT had the best performance, answering 82.4% of questions correctly, followed by humans (75%) and ChatGPT-3.5 (65.9%) [47]. Tao BK et al. noted that Bing Chat answered 73.6% of 913 BCSC-SAP questions correctly in August 2023, against 59.69% of correctly answered questions by ChatGPT-3.5 [42]. Lin et al. also noted significant improvements from ChatGPT-3 to ChatGPT-4 in 260 questions from BCSC-SAP, where ChatGPT-4 attained higher scores than human users and would be classified as a passing score [48].

Fowler et al. found ChatGPT-4.0 significantly outperformed Google Bard on Fellowship of the Royal College of Ophthalmologists (FRCOphth) Part 1 exam questions, obtaining better results than the historical human pass marks [4]. Raimondi et al. compared multiple LLMs on FRCOphth exam questions, with ChatGPT-4.0 and Bing Chat (which is also powered by GPT-4) showing a significantly higher accuracy than ChatGPT-3.5 and Google Bard [70]. Thirunavukarasu AJ et al. compared GPT-3.5, GPT-4, PaLM 2, LLaMA, expert ophthalmologists, and doctors in training in a mock examination based on questions used for training to FRCOphth exams. In their study, ChatGPT-4.0 performance was superior to all other LLMs and unspecialized junior doctors, while it was comparable to expert ophthalmologists and ophthalmology residents [59]. 

Alexander et al. evaluated ChatGPT’s performance on some of the Indian Foreign Medical Graduate Examination multiple-choice and short-answer questions in clinical ophthalmology, with both versions achieving high scores (80% or more) in all areas tested, however presenting some incorrect recommendations for conjunctivitis management and cataract diagnosis, two fairly common ophthalmologic conditions. The prompts were given in English [12].

Gobira et al. evaluated ChatGPT-3.5 on the Brazilian Council of Ophthalmology Board Examination, where only 41.6% of the questions were answered correctly, noting a particularly low accuracy in mathematical and clinical questions. ChatGPT-3.5 was not able to attain a passing grade on this test [9]. Sakai D et al. assessed ChatGPT-3.5 and 4.0 on the Japanese Ophthalmology Society board examinations, using prompts written in Japanese. ChatGPT-3.5 answered around 22% of questions correctly, and ChatGPT-4.0 was correct in 46% of questions. Both models presented significantly inferior performance to average human examinees and did not meet the marks of the models in other languages [63]. These findings might be due to differences in the complexity and evaluation of questions from the Japanese Ophthalmology Society and Brazilian Council of Ophthalmology board examinations, but most likely reflect disparities in the accuracy of the models across different languages.

Moshirfar M et al. studied ChatGPT-4.0, ChatGPT-3.5, and average human responses on StatPearls questions, reporting that ChatGPT-4.0 presented a significantly higher accuracy than its ChatGPT-3.5 and the average human response accuracy. ChatGPT’s performance was also tested specifically in questions applied to clinical cases, which require higher level thinking and interpretation of multiple inputs of clinical information [71]. Huang et al. found ChatGPT-4.0 outperformed glaucoma and retina specialists in diagnostic accuracy and completeness in responses to clinical cases and frequently asked questions from AAO [72]. Jiao C et al. highlighted the advancements of ChatGPT-4.0 over 3.5 in ophthalmic knowledge by comparing the performance in answering questions from clinical cases available at the AAO’s “Diagnose This” question bank. Most significant improvements were noted especially in neuro-ophthalmology and image-related questions [8].

Inayat H et al. compared ChatGPT’s performance with ophthalmology residents and staff consultants in determining diagnosis and urgency in custom questions based on real cases commonly presented in an ophthalmologic emergency setting, noting a high concordance with human practitioners for diagnosis, but not for definition of urgency [61].

In October 2023, Mihalache et al. published a new study evaluating ChatGPT’s capability of interpreting multimodal and imaging input using OCTCases, a medical education platform based out of the Department of Ophthalmology and Vision Sciences at the University of Toronto. ChatGPT answered 70% of the questions correctly, having a better performance in text-based questions (82% of answers were correct) and in retinal disease cases (77% of answers were correct) when compared to other specialties [54].

Milad D et al. discussed GPT-4’s capability to diagnose and manage complex ophthalmologic cases in both open-ended and multiple-choice questions based on Journal of the American Medical Association Ophthalmology’s Clinical Challenges, in which a limited accuracy was observed. The model was capable of correctly diagnosing 48% of the cases, and suggested adequate first steps of management in 63% of the cases [11].

In summary, while GPT-4 and other LLMs show significant potential in medical education, their performance varies with the complexity and specificity of the information requested. Variance was also observed between simple fact recalling and complex data interpretation and decision making, and also across different languages. A clear trend of continuous improvement has been seen, both through new capabilities such as real-time internet access or multimedia input availability, and through comparisons of ChatGPT-4.0 and 3.5 against multiple benchmarks. Nonetheless, further improvements are necessary to enhance accuracy and reliability, particularly in specialized fields such as retinal care. While ChatGPT can be applied as a useful and flexible learning assistance tool, all information obtained should be fact-checked and compared with updated medical literature.

### Patient counseling

Patient counseling with ChatGPT was portraited in the studies in two main forms: patients independently seeking information from LLMs and healthcare providers using LLMs to create educational materials for their patients. Each method comes with its own set of benefits and challenges.

A study evaluating ChatGPT’s responses to common vitreoretinal disease questions found that only 15.4% of the chatbot’s answers were completely accurate. Moreover, the responses were inconsistent, with 50% showing material changes when the same questions were asked two weeks later, and in some cases, the accuracy worsened. For example, ChatGPT incorrectly suggested injection therapy and laser treatment for an epiretinal membrane, which could mislead patients and potentially cause harm [17].

Similarly, when ChatGPT was used to answer questions about “floaters,” a common patient concern, the chatbot provided general information but failed to emphasize the urgency of consulting an ophthalmologist, which is critical given that floaters can be a sign of retinal detachment—a condition that requires immediate medical attention. The study also highlighted that ChatGPT’s language complexity is above the average reading level of many patients, potentially limiting its accessibility [23].

In addition to specific condition-related queries, other studies focused on the quality, empathy, and safety of ChatGPT’s responses to common retina patient questions. Expert-edited LLM responses were found to potentially improve the quality and empathetic tone of patient communications, but the need for human oversight to ensure accuracy and safety was emphasized. Similarly, ChatGPT-4’s recommendations for ophthalmology-related questions were mostly appropriate, though there was significant variation across subspecialties, necessitating further optimization before clinical use [25, 73].

Momenae et al. compared ChatGPT-3.5 and ChatGPT-4 in generating responses about retinal surgeries, finding ChatGPT-4 more accurate, with appropriateness rates of 84.6% for retinal detachments, 92% for macular holes, and 91.7% for epiretinal membranes. However, its responses were considered difficult to understand, requiring a college-level education, highlighting the need for improvements to make AI-generated medical advice both accurate and accessible to patients [74, 75]. In another study, Wu et al. evaluated the ability of ChatGPT to educate diabetic patients about diabetic retinopathy using a keyword-based scoring system. ChatGPT provided basic but incomplete answers, scoring poorly on key terms like macular edema [76].

Moreover, one study evaluated ChatGPT’s performance in providing information about retinal diseases and uveitis. While generally accurate, the study emphasized the need for rigorous evaluation to ensure high standards of accuracy and reliability [77]. A comparison of ChatGPT 3.5, Bing AI, and Google Bard in addressing common questions from patients with age-related macular degeneration (AMD) showed that ChatGPT 3.5 consistently outperformed the other models, particularly excelling in technical queries [19]. ChatGPT’s ability to diagnose and provide prehospital management recommendations for urgent eye conditions was also assessed, showing high triage accuracy but highlighting the potential for harmful advice, thus underscoring the need for continuous improvement [78].

Cheong et al. compared the performance of generative (ChatGPT-4, ChatGPT-3.5, and Google Bard) and retrieval-based (OcularBERT) chatbots in answering patient questions regarding age-related macular degeneration (AMD) and diabetic retinopathy (DR). ChatGPT-4 and ChatGPT-3.5 outperformed the other models in both accuracy and quality of responses, demonstrating their potential to answer domain-specific medical questions. The study underscored that these generative chatbots are capable of accurately addressing domain-specific questions outside their initial training [79].

LLMs can assist physicians in generating educational materials, enhancing the quality and efficiency of patient education. One study investigated ChatGPT’s ability to simplify healthcare information from the American Academy of Ophthalmology (AAO), finding significant improvements in the readability of articles in Spanish but limited impact on articles in English [80]. Another study evaluated ChatGPT, Bing AI, and Docs-GPT in providing responses to common patient queries about virtreoretinal surgery. The study found that while all three LLMs generally provided accurate and sufficient information, Bing AI performed the best overall [15]. Cappellani F et al. highlighted ChatGPT’s ability to provide valuable patient education, although it still provided incorrect or incomplete information that might be harmful for patients [14].

The effectiveness of ChatGPT and Bard in generating patient-targeted health information about uveitis was also evaluated, with ChatGPT producing more readable content, highlighting its potential to improve health literacy [81]. Furthermore, ChatGPT’s capability to swiftly generate discharge summaries and operative notes across various subspecialties has been demonstrated. While human oversight is required to customize and enhance these outputs, this approach substantially reduces the time spent on documentation and improves overall patient care [31].

A study comparing ChatGPT, Bing Chat, and WebMD Symptom Checker found that ChatGPT-4 provided highly accurate and detailed information, comparable to that of ophthalmology trainees, suggesting its potential as a valuable tool for patient self-triage [28]. Another study compared Bard and ChatGPT’s responses to ophthalmology-related prompts, finding both to have significant potential but also limitations that need addressing [60].

Nanji et al. evaluated the quality of postoperative ophthalmology instructions provided by ChatGPT, Google Search, and institutional handouts from Canadian and U.K. sources. The study found that while ChatGPT’s instructions contained procedure-specific information comparable to the other sources, they were generally less understandable, particularly when compared to the U.K. institution’s materials. This study, however, did not specifically request an accessible response [18].

Bernstein et al. explored the capability of ChatGPT to provide ophthalmology advice in comparison to human ophthalmologists. The study revealed that the chatbot’s responses were comparable to those written by ophthalmologists regarding the inclusion of incorrect information, potential harm, and adherence to medical consensus. However, the chatbot was more likely to be identified as AI-generated by expert reviewers, though with only moderate accuracy. The study highlights the potential for LLMs to assist in patient education but also underscores the risks, such as “hallucinations” or generating plausible yet inaccurate information​ [16].

### Triage and Pre-hospital management

Recent studies have explored the potential of ChatGPT in triaging ophthalmic symptoms and offering pre-hospital management advice. Knebel et al. demonstrated that ChatGPT achieved a high triage accuracy of 93.6% when evaluating acute ophthalmological symptoms using fictional case vignettes. Despite its general effectiveness, the study highlighted that 32% of the responses carried the potential to cause harm, underscoring the need for continuous improvement and oversight in using AI for medical triage [78].

Gopalakrishnan et al. also reported that ChatGPT provided highly accurate triage recommendations but often delivered general and vague treatment advice, pointing to the necessity for greater specificity in its responses [82]. Similarly, Lyons et al. compared ChatGPT’s performance with that of ophthalmology trainees, finding that ChatGPT accurately listed the correct diagnosis among the top three in 93% of cases and provided appropriate triage recommendations in 98% of cases. The study concluded that ChatGPT outperformed Bing Chat and WebMD Symptom Checker, suggesting its value in patient self-triage and initial assessments [28].

The advancements of GPT-4 over its predecessor GPT-3.5 were further illustrated by Waisberg et al., who noted that GPT-4 addressed previous shortcomings in specific scenarios, such as macular degeneration, where it now recommends immediate medical attention for severe or sudden visual changes. This improvement underscores GPT-4’s enhanced problem-solving abilities and its broader knowledge base [26].

Despite these advancements, a study by Inayat et al. compared the performance of ChatGPT to ophthalmology residents and staff consultants using a training tool based on real on-call pages. While residents and staff performed better in overall accuracy, ChatGPT exhibited strong diagnostic capabilities but showed inconsistency in triage, often favoring more urgent assessments compared to human experts. The findings suggest that, while ChatGPT has potential as an educational resource, human decision-making remains crucial in medical triage due to the nuanced nature of clinical management [61].

The incorporation of LLMs like GPT-4 in triaging ophthalmic symptoms marks a significant step forward in healthcare, offering the potential to improve the accuracy and efficiency of initial patient assessments. These models can match or even exceed the diagnostic accuracy and triage appropriateness of trained ophthalmology professionals in certain scenarios. However, the risk of incorrect or harmful advice emphasizes the need for ongoing refinement and rigorous validation of these AI systems before they can be reliably used in real-world clinical settings. Future developments should focus on enhancing model reliability and integrating advanced features like image analysis to further improve the utility of LLMs in medical triage.

## Conclusion

ChatGPT and other LLMs offer considerable potential in transforming various aspects of retinal healthcare, from screening and diagnosing to disease management and patient education. Their ability to assist in decision-making, triage, and generating educational materials presents a promising opportunity to enhance clinical workflows and patient care. Notably, there has been a clear improvement from version 3.5 to version 4, reflecting ongoing advancements in AI technology. However, these tools are not without limitations. Their current performance, particularly in scenarios involving figure-based inputs, and the need for constant supervision highlight the importance of cautious integration into clinical practice.

To maximize the effectiveness of AI in ophthalmology, it is crucial to ensure that the information generated by these models is accurate, relevant, and comprehensible. The issue of generating understandable outputs is particularly nuanced, often depending on how prompts are crafted and specified. Studies that have reported less understandable outputs may not have explicitly requested accessible responses, underscoring the importance of prompt engineering. Additionally, the consistent need for human supervision cannot be overstated. While AI can provide valuable support, it is crucial to ensure that the final decision-making remains in the hands of healthcare professionals. One effective strategy to improve accuracy is to prompt the AI to generate a list of diagnoses or options rather than a single definitive answer. This approach enhances the reliability of the information provided, allowing clinicians to make the final informed decisions.

Looking forward, advancements in AI should aim to overcome these challenges, focusing on refining models to better meet the needs of both patients and healthcare providers. By addressing these issues, we can fully harness the potential of AI in retinal healthcare, ultimately improving diagnostic accuracy, disease management, and patient education.

## Electronic supplementary material

Below is the link to the electronic supplementary material.

Supplementary Material 1

Supplementary Material 2

## Data Availability

The datasets generated and/or analysed during the current study, as well as the review registration and prospectus, are available in the Open Science Framework (OSF) repository, https://osf.io/nrjt7. The prospectus is also included in this article’s supplementary material.

## Abbreviations

AAO: American Academy of Ophthalmology
ADA: Advanced Data Analysis
AMD: Age–related Macular Degeneration
AI: Artificial Intelligence
BCSC: SAP–Basic and Clinical Sciences Course’s Self–Assessment Program
CA: California (USA)
DALK: Deep Anterior Lamellar Keratoplasty
DME: Diabetic Macular Edema
DR: Diabetic Retinopathy
FMGE: Foreign Medical Graduate Examination
FRCOphth: Fellowship of the Royal College of Ophthalmologists
GPT: Generative Pretrained Transformer
ICD: International Classification of Diseases
ICGA: Indocyanine Green Angiography
IOL: Intraocular Lens
LLM: Large Language Model
MD: Macular Degeneration
ML: Machine Learning
OKAP: Ophthalmology Knowledge Assessment Program
OCT: Optical Coherence Tomography
OSF: Open Science Foundation
PK: Penetrating Keratoplasty
PRISMA: Preferred Reporting Items for Systematic Reviews and Meta–Analyses
USA: United States of America
WI: Wisconsin (USA)
