# Supplementary material for: Applications of ChatGPT in the diagnosis, management, education, and research of retinal diseases: a scoping review
Source: Int J Retina Vitreous. 2024 Oct 17;10:79. doi: 10.1186/s40942-024-00595-9 (PMC11487877; doi:10.1186/s40942-024-00595-9)
Supplement: Supplementary file 2 — Supplementary Material 2 [file 40942_2024_595_MOESM2_ESM.pdf]

OSF REGISTRIES Add New My Registrations Help Donate

Applications of ChatGPT in the Diagnosis, Management and Research of Retinal Diseases: a Scoping Review

Public registration Updates

Metadata

verview
etadata
les
esources
iki
omponents 0
nks 0
nalytics
omments 0

n practice
urces
Data
Analytic code
Materials
Papers
Supplements

Landing Page

Intended use

This Generalized Systematic Review Registration Form is intended as a general-purpose registration form. The form is designed to be applicable to reviews across disciplines (i.e., psychology, economics, law, physics, or any other field) and across review types (i.e., scoping review, review of qualitative studies, meta-analysis, or any other type of review). That means that the reviewed records may include research reports as well as archive documents, case law, books, poems, etc. This form, therefore, is a fall-back for more specialized forms and can be used if no specialized form or registration platform is available. Below are some currently available specialized registration tools you may consider:

Specialized registration platforms

PROSPERO is a free database of health-related systematic review protocols for health-related outcomes.

Specialized guidance

Consider using the following guidelines when completing your registration:

The Non-Interventional, Reproducible, and Open (NIRO) Systematic Reviews guideline, which includes fields specific to non-interventional reviews: https://osf.io/f3brw/

Methodological Expectations of Cochrane Intervention Reviews (MECIR): CID: 20.500.12592/vxj0sb

Methodological Expectations of Campbell Collaboration Intervention Reviews (MECCIR): https://www.campbellcollaboration.org/meccir.html

Preferred Reporting Items for Systematic reviews and Meta-Analysis Protocols (PRISMA-P): https://doi.org/gcpzzq

Preferred Reporting Items for Systematic reviews and Meta-Analyses literature Search extension (PRISMA-S): https://doi.org/gh2z2k

Peer Review of Electronic Search Strategies (PRESS): https://doi.org/10.1016/j.jclinepi.2016.01.021

Relation to reporting guidelines

Many disciplines have developed reporting guidelines for specific types of reviews (e.g., ROSES: the RepOrting standards for Systematic Evidence Syntheses in environmental research, and PRISMA: the Preferred Reporting Items for Systematic Reviews and Meta-Analyses). Whereas reporting guidelines were optimized for application after conclusion of a systematic review, this form was optimized to publicly register ('freeze') the research plans (or to record adjustments to research plans) before (or during) a systematic review. These different end goals resulted in different choices regarding included items. For example, this form includes a number of questions about planning that are important for a registration but typically are not included in reporting guidelines.

Nonetheless, these reporting guidelines do partly capture the same information as registration forms. For each item in this form, we specified the corresponding PRISMA item (PRISMA items P1-P22 and P25-27 were applicable; P16-P23 cover reporting of results and P24 refers to registration forms like this). Researchers planning to use a specific reporting standard to report the results of their review, should enter the information required by that reporting standard in the corresponding (overarching) fields of this form.

Instructions for effectively using the form

To align with general use and open science best practice, all items are mandatory. Completion makes your registration more useful for readers, funders, and others, so check carefully whether you did not accidently omit an item. If an item asks about a procedure you do not plan to use or is not applicable, indicate that in the corresponding field (including, ideally, the underlying reason).

You should be transparent about any deviations from the preregistration and provide the rationale for these deviations in your final review. If you already foresee some deviations when filling out the form (e.g., you anticipate that you will not have enough studies in a moderator group), provide a contingency plan for these deviations in the relevant parts of the registration.

Planned improvements / extensions

The aim of this registration form is to be optimally inclusive (i.e., to be usable for registration of any systematic review, regardless of scientific discipline or review type). Because this aim precludes 1:1 correspondence with the existing reporting guidelines, this form is also intended as a basis to develop more specialized forms that do correspond closely to more specific reporting guidelines. Such specialized forms can include, for example, additional fields, added comments, and worked examples. Please contact the Center of Open Science at contact@cos.io if you would like to propose such a specialized version. Please do reach out if you want to be involved in any of these projects.

Citation

Van den Akker, O. R., Peters, G. Y., Bakker, C., Carlsson, R., Coles, N. A., Corker, K. S., Feldman, G., , Moreau, D., Nordström, T., Pickering, J. S., Riegelman, A., Topor, M., Veggel, N., Yeung, S., Mellor, D., & Pfeiffer, N. Generalized Systematic Review Registration Form. MetaArXiv.. https://doi.org/g5fj.

Review Methods

In this section, you register the general type, background and goals of your review.

Type of review

This scoping review will adhere to the PRISMA (Preferred Reporting Items for Systematic reviews and Meta-Analyses) guidelines for scoping reviews, adapting these recommendations to thoroughly explore the breadth and depth of the applications of ChatGPT within retinal healthcare.

Review stages

Search, Pilot Screening, Screening, Extraction, Synthesis.

Current review stage

Screening

Start date

April 8th 2024.

End date

May 26th 2024

Background

Retinal diseases are among the leading causes of visual impairment worldwide. The complexity of these conditions requires precise diagnostic tools and effective management strategies. The advent of artificial intelligence, particularly conversational AI models like ChatGPT, has opened new avenues for innovation in this field. This review aims to synthesize the current knowledge on the applications of ChatGPT in retinal disease care and research, identifying its potential benefits and limitations.
A preliminary search of PROSPERO International prospective register of systematic reviews, MEDLINE, the Cochrane Database of Systematic Reviews and JBI Evidence Synthesis was conducted and no current or underway systematic reviews or scoping reviews on the topic were identified.

Primary research question(s)

Objectives
- To evaluate the existing applications of ChatGPT in the diagnosis of retinal diseases.

Metadata
Contributors
Victor C. F. Bellanda
Description
Show more
Registration type
Generalized System
Date registered
April 28, 2024
Date created
April 28, 2024
Associated projec
osf.io/9cyxw
Internet Archive
https://archive.org/
Category
Project
Registration DOI
https://doi.org/10.1
Publication DOI
No publication DOI
Subjects
Ophthalmology
Affiliated institut
This registration ha
License
No license
Tags
Artificial Intelligen
Citation
osf.io/nrjt7

- To assess the potential roles of ChatGPT in the management and treatment decision processes for retinal conditions.
- To explore the utilization of ChatGPT in facilitating research into retinal diseases.
- To evaluate the applications of ChatGPT in other aspects of delivering healthcare, such as organizing patient notes or preparing handouts and educational material related to retinal diseases and their treatments.

Conceptual Framework
We will utilize the Population, Exposure, Outcome (PEO) model to structure our research question and guide our selection criteria:
Research Question: What are the existing applications of ChatGPT across all aspects of retinal healthcare?
Population: The review will consider studies involving researchers, healthcare professionals, patients, and their families within the care setting for retinal diseases.
Exposure: The exposure of interest is the use of ChatGPT in any healthcare delivery step concerning retinal diseases. This encompasses (but is not limited to) research, diagnosis, management, medical information handling, charting, coding, billing, and patient or professional education.
Outcome: The outcomes of interest include the feasibility of using ChatGPT, its effectiveness in facilitating healthcare-related tasks, the accuracy of information provided, and the limitations or challenges encountered in its application.

Secondary research question(s)

1- What are the difficulties and limitations in using ChatGPT for assisting in retinal care?
2- How can we explore the ethical considerations involved?

Expectations / hypotheses

The eligible articles will refer mostly to one or more of the following applications of ChatGPT in retinal care:
1 - Academics and research
2 - Charting
3 - Coding and billing
4 - Diagnosis
5 - Disease management
6 - Literature consulting
7 - Medical education
8 - Patient counseling

The results will be synthesized narratively to map the scope of ChatGPT's applications in retinal healthcare and to identify patterns, themes, potentials and limitations, and gaps in the literature. A narrative synthesis will facilitate an understanding of how ChatGPT is being utilized, its benefits, and its drawbacks in the field of retinal disease.

Dependent variable(s) / outcome(s) / main variables

The outcomes of interest include the feasibility of using ChatGPT, its effectiveness in facilitating healthcare-related tasks, the accuracy of information provided, and the limitations or challenges encountered in its application.

Independent variable(s) / intervention(s) / treatment(s)

Using ChatGPT in any healthcare delivery step concerning retinal diseases. This encompasses (but is not limited to) research, diagnosis, management, medical information handling, charting, coding, billing, and patient or professional education.

Additional variable(s) / covariate(s)

No additional variables.

Software

Two independent reviewers will screen the titles and abstracts of identified studies for eligibility based on the inclusion and exclusion criteria. Full texts of potentially eligible studies will be retrieved and independently assessed. Disagreements will be resolved by consensus or involvement of a third reviewer. The process will be developed using the platform Rayyan, a free web and mobile app publicly available at http://rayyan.ai.

Funding

This study is self-funded, and no external direct financial support was received. The authors contribute their time voluntarily and pay for their own equipments, physical space, supplies and general services needed to conduct all activities related to accessing study materials, compiling and analyzing data, and writing and reviewing this manuscript. External support is indirectly received from the University of São Paulo, a public university in Brazil with no conflicts of interest in the area, in the form of free access to multiple journals and scientific archives.

Conflicts of interest

The authors declare having no conflicts of interest pertaining to the theme of this study.

Overlapping authorships

No overlapping authorships are expected for this study.

Search Strategy

In this section, you register your search strategy: the procedures you designed to obtain all (potentially) relevant sources to review (e.g., articles, books, preprints, reports, case law, policy papers, archived documents).

Databases

PubMed, Scopus, MEDLINE, Embase.

Interfaces

PubMed, Elsevier (Scopus and Embase), Virtual Health Library (MEDLINE).

Grey literature

Hand-searching of reference lists and grey literature will also be performed to ensure comprehensive coverage of the topic. This includes, but is not limited to, searching web-based search engines (eg, Google Scholar) and repositories (eg, ResearchGate). Preprints included in the Scopus repository will also be considered. Scopus covers preprints from the following repositories: arXiv, ChemRxiv, bioRxiv, medRxiv, SSRN, TechRxiv, and Research Square.

Inclusion and exclusion criteria

Conceptual Framework
We will utilize the Population, Exposure, Outcome (PEO) model to structure our research question and guide our selection criteria:
Research Question: What are the existing applications of ChatGPT across all aspects of retinal healthcare?
Population: The review will consider studies involving researchers, healthcare professionals, patients, and their families within the care setting for retinal diseases.
Exposure: The exposure of interest is the use of ChatGPT in any healthcare delivery step concerning retinal diseases. This encompasses (but is not limited to) research, diagnosis, management, medical information handling, charting, coding, billing, and patient or professional education.
Outcome: The outcomes of interest include the feasibility of using ChatGPT, its effectiveness in facilitating healthcare-related tasks, the accuracy of information provided, and the limitations or challenges encountered in its application.

Criteria for selecting articles for this review
We will include articles that:
- Discuss the application of ChatGPT across all aspects of retina healthcare and knowledge production.
- Involve the population outlined in the PEO model.
- Evaluate the outcomes based on the feasibility, facilitation of tasks, accuracy, and/or limitations of using ChatGPT.

Articles will be excluded if they:
- Are not related to retinal diaseases.
- Assess other forms of AI with similar capabilities to ChatGPT, including other forms of Large Language Models (LLM), without directly comparing them with ChatGPT.
- Are purely theoretical without empirical evaluation or application.

- Are not available in full text or not in English, Portuguese, Spanish, French, German, or Italian.

Query strings

PubMed: (("ChatGPT"[All Fields] OR "GPT"[All Fields] OR "LLM"[All Fields] OR "large language"[All Fields]) AND ("ophthalmologie"[All Fields] OR "ophthalmology"[MeSH Terms] OR "ophthalmolog*"[All Fields] OR "ophthalmology s"[All Fields] OR "retin*"[All Fields] OR "vitre*"[All Fields] OR "uvei*"[All Fields] OR "uvea*"[All Fields] OR "chor*"[All Fields] OR "macul*"[All Fields]) AND 2022/01/01:2024/12/31[Date - Publication]) AND (2022/1/1:2024/12/31[pdat])

Scopus: TITLE-ABS-KEY ({ChatGPT}) OR TITLE-ABS-KEY ({GPT}) OR TITLE-ABS-KEY ({LLM}) OR TITLE-ABS-KEY ({large language}) AND TITLE-ABS-KEY (ophthalmology) OR TITLE-ABS-KEY (retin*) OR TITLE-ABS-KEY (vitre*) OR TITLE-ABS-KEY (uvea*) OR TITLE-ABS-KEY (uvei*) OR TITLE-ABS-KEY (chor*) OR TITLE-ABS-KEY (macul*) AND PUBYEAR > 2021 AND PUBYEAR < 2025 AND (LIMIT-TO (LANGUAGE , "English") OR LIMIT-TO (LANGUAGE , "French") OR LIMIT-TO (LANGUAGE , "Spanish") OR LIMIT-TO (LANGUAGE , "Portuguese") OR LIMIT-TO (LANGUAGE , "Italian") OR LIMIT-TO (LANGUAGE , "German"))

MEDLINE: (chatgpt OR gpt OR "large language") AND (ophthalmolog* OR retin* OR uvea* OR uvei* OR chor* OR vitre* OR macul*) AND (la:("en" OR "de" OR "es")) AND (year_cluster:[2022 TO 2024])

Embase: ((ChatGPT OR GPT OR LLM OR 'large language') AND (ophthalmology/exp OR ophthalmolog* OR retin* OR vitre* OR uvei* OR uvea* OR chor* OR macul*) AND 2022/de) AND (2022/de)

Search validation procedure

No specific search validation procedures are planned for this review.

Other search strategies

No other specific search strategies are planned for this review.

Procedures to contact authors

Authors will be contacted only if access to the full text of potentially eligible articles is not possible through the available libraries and repositories. Should they happen, contacts will pertain solely to the request for access to the texts. Meta data about all communications will be disclosed in the final report.

Results of contacting authors

Meta data about all communications will be disclosed in the final report.

Search expiration and repetition

No repetitions are planned.

Search strategy justification

The search strategy for this review was meticulously crafted through a collaborative and iterative process involving all authors, who are experient in database searching and subject matter experts in ophthalmology. We embarked on initial searches across various platforms, reviewing the relevancy of results to ensure our approach was sound. Our collective analysis led to the refinement of search strings, honing in on those yielding the highest percentage of relevant articles.

For our search to be comprehensive yet focused, we stipulated that each article must contain at least one keyword from both of our designated groups. Group 1 keywords are centered around the technology in question: ChatGPT, GPT, LLM, or "large language". Group 2 keywords pivot around the field of retinal diseases: ophthalmolog*, retin*, vitre*, uvea*, uvei*, chor*, and macul*.

Initially, "eye" was a part of Group 2, and "generative" was included in Group 1. However, upon review, "eye" was casting too wide a net, capturing an excess of articles not specific to ophthalmology. Similarly, "generative" was drawing in a significant number of irrelevant results related to generative adversarial networks, rather than our intended AI models. Therefore, we refined our terms to the most pertinent and specific to our research. This strategic approach strikes a balance between the comprehensive scope and methodological stringency, acknowledging the constraints of practicality while not compromising scientific rigor.

Miscellaneous search strategy details

To ensure comprehensive coverage of the topic, reference lists and grey literature will also be hand-searched. This includes, but is not limited to, searching web-based search engines (e.g., Google Scholar) and repositories (e.g., ResearchGate).

Screening

In this section, you register your screening procedure: the procedure you designed to eliminate all irrelevant sources from the results of the search strategy (and retain the relevant sources).

Screening stages

Article deduplication will be conducted using the Rayyan deduplication tool, which automatically removes duplicate copies of articles sharing the same DOI. Subsequently, Rayyan will highlight articles with similar titles, abstracts, or keywords for the authors to review and confirm whether they represent duplicates. Following deduplication, the screening will be conducted in a single round, examining titles, abstracts, and keywords. This process will be independently and blindly carried out by two reviewers.

Screened fields / blinding

Titles, abstracts, authors, and journal names will be visible to the screeners.

Used exclusion criteria

Articles that are excluded will be classified based on the following exclusion criteria:
- not GPT
- not retina
- unsupported language
- text not available

Screener instructions

associated OSF project contains these instructions.
Screening instructions are the review protocol itself. No additional documents were elaborated providing specific instructions.

No files selected

Screening reliability

Independent, blind screening will be warranted by the Rayyan platform. Results will only be unblinded after all articles were screened and received a final classification.

Screening reconciliation procedure

Disagreements will be resolved by discussion and consensus or involvement of a third reviewer.

Sampling and sample size

All sources included through the screening procedure will be used in the final analysis.

Screening procedure justification

Two independent reviewers will screen the titles and abstracts of identified studies for eligibility based on the inclusion and exclusion criteria. They will then retrieve and independently assess the full texts of potentially eligible studies. Disagreements will be resolved by consensus or the involvement of a third reviewer. We believe this is sufficient to establish a rigorous inclusion of relevant articles.

Data management and sharing

The sources obtained from the database searches will be disclosed with the final article in the format of supplementary files, available for everyone. File formats are RIS (Scopus, MEDLINE, Embase) and PubMed TXT (PubMed).

Miscellaneous screening details

No further details.

Extraction

In this section, you register your plans for data extraction: the procedures you designed to extract the data you are interested in from the included sources. Examples of such data are text fragments, effect sizes, study design characteristics, year of publication, characteristics of measurement instruments, final verdicts and associated penalties in a legal system, company turnovers, sample sizes, or prevalences.

Entities to extract

Each study that is potentially eligible will be categorized into one or more of the following areas:

1 - Academics and research
2 - Charting
3 - Coding and billing
4 - Diagnosis
5 - Disease management
6 - Literature consulting
7 - Medical education
8 - Patient counseling
9 - Other

Qualitative data fragments deemed relevant and general highlights from each study will be extracted from each article and included in a database common to both reviewers.

Extraction stages

Extraction will be done in a single round by two extractors working in parallel.

Extractor instructions

The extractors will iclude highlights and fragments of each article into a common database. Extraction instructions are the review protocol itself. No additional documents were elaborated providing specific instructions.

No files selected

Extractor masking

No masking will be used for the extraction process.

Extraction reliability

The extraction process will not be independent. The reviewers may discuss relevant topics from each article and determine which elements will be incorporated in the common database.

Extraction reconciliation procedure

Not applicable.

Extraction procedure justification

Considering this scoping review aims at gathering information on common uses of ChatGPT in the retina healthcare and research settings, and not elaborating guidelines or combining study results, we considered the open, common extraction procedure to be sufficient and adequate.

Data management and sharing

The results will be synthetized in the form of an academic article to be published in a scientific journal. No sharing of intermediate documents or drafts is planned.

Miscellaneous extraction details

No further details.

Synthesis and Quality Assessment

In this section, you register the procedure for the review's synthesis: the procedure you designed to use the data that was extracted from each source to answer your research question(s). This often includes transforming the raw extracted data, verifying validity, applying predefined inference criteria, interpreting results, and presenting results. Additionally, you register procedures you designed to assess bias in individual sources and the synthesis itself.

Planned data transformations

Each study that is potentially eligible will be categorized into one or more of the following areas:

1 - Academics and research
2 - Charting
3 - Coding and billing
4 - Diagnosis
5 - Disease management
6 - Literature consulting
7 - Medical education
8 - Patient counseling
9 - Other

Qualitative data fragments deemed relevant will be extracted from each article and included in a database common to both reviewers. The original draft of information extracted from the articles will be synthesized into a common text. It is beyond the scope of this review to verify the validity of each article individually. No other data transformation is planned.

Missing data

Only data that are present in the articles will be considered in the final review.

Data validation

Not applicable.

Quality assessment

Not applicable.

Synthesis plan

A synthesis will be elaborated for each one of the previously mentioned areas (1 to 9). In parallel, ethical aspects and limitations will also be discussed within each item.

Criteria for conclusions / inference criteria

Unless otherwise specified by the authors, all eligible articles will be included in their respective sessions of the final report. When authors exclude an article, they must specify the reasons in the final text.

Synthesist blinding

Not applicable.

Synthesis reliability

Two synthetists will work together in an collaborative review.

Synthesis reconciliation procedure

Not applicable.

Publication bias analyses

Not applicable.

Sensitivity analyses / robustness checks

Not applicable.

Synthesis procedure justification

We believe that, as a scoping review aiming to elaborate on the different uses of ChatGPT in retinal care, a thorough review of each eligible article and a collaborative synthesis of findings by two reviewers should be adequate to generate the final analysis.

Synthesis data management and sharing

The results will be presented in the form of an academic article to be published in a scientific journal. No sharing of intermediate documents or drafts is planned.

Miscellaneous synthesis details

No further details.

Copyright © 2011-2024 Center for Open Science | Terms of Use | Privacy Policy | Status | API
TOP Guidelines | Reproducibility Project: Psychology | Reproducibility Project: Cancer Biology
